# Applicant Ranking Criteria in Adult Reconstruction Fellowship: Your Interview Counts When Applying for Fellowship

**DOI:** 10.7759/cureus.20636

**Published:** 2021-12-23

**Authors:** Shyam A Patel, Jillian Glasser, Ellis M Berns, Caitlin C Barrett, Derek Jenkins, Valentin Antoci, Eric Cohen, John Froehlich

**Affiliations:** 1 Orthopaedics, Rhode Island Hospital, Brown University, Providence, USA; 2 Adult Reconstruction, University Orthopedics, Providence, USA; 3 Orthopaedics, Warren Alpert Medical School, Brown University, Providence, USA

**Keywords:** continued medical education, interview, adult reconstruction, arthroplasty, fellowship match

## Abstract

Background

The criteria for successful ranking in the fellowship match remains unclear. Although some data are available regarding the qualities sought after in medical students for the residency match, little information is available regarding the fellowship match. In this study, for arthroplasty applications to our institution, the interview was hypothesized to be the most important factor for ranking, with little impact from other commonly assessed variables.

Methodology

All 40 applicants who applied for fellowship were selected to interview for the 2017 Brown University Comprehensive Adult Reconstruction Fellowship and were evaluated on the interview, United States Medical Licensing Examination (USMLE) scores, letters of recommendation, personal statement, extracurricular activities, research, and caliber of undergraduate institution, medical school, and residency program. The interview score was based on a combined score of individual components of personality, program fit, and research.

Results

Of the 40 applicants who were interviewed, eight did not match. The interview score was the only statistically significant variable and had the highest correlation with ranking (r = 0.92). Moreover, extracurricular activities correlated with a higher ranking whereas USMLE Step 1/Step 2 scores had a relatively low correlation (r = 0.32 and 0.29, respectively). Recommendation letters and caliber of medical school, undergraduate education, and residency demonstrated low correlations. The personal statement and research components had the lowest correlations.

Conclusions

The combined interview score, in particular the personality and program fit components, was the most important determinant of successful ranking at our institution. Because all 40 applicants who applied for an arthroplasty fellowship at our institution were selected for an interview, there was no pre-interview selection bias that would confound these results. However, the ranking does not correlate with an applicant’s success in fellowship, and further research is required to determine the qualifications of a successful surgeon.

## Introduction

Candidate selection during medical training remains a controversial subject. From medical school to fellowship, candidates are assessed using conventional criteria that function as markers for what selection committees predict will yield successful physicians and/or surgeons. Noticeable incongruities exist among criteria used across stages in training, specialty/subspecialty, and programs. Furthermore, the definition of what is considered “successful” is both subjective and variable [1]. Recently, research has further elucidated the process of selection for orthopedic residency; however, little has been published on the subject of fellowship selection [1,2].

Not surprisingly, the fellowship process has evolved to resemble the residency match [3]. The pathway now involves a formalized match in which both programs and fellowship directors have more information regarding the other party than they did in years past [4]. Although there is a higher match rate (85%) for orthopedic fellowships on the whole compared to orthopedic residency (65%), more than 90% of orthopedic residents pursue a fellowship. Consequently, a growing number of orthopedic residents applying for a limited number of fellowship positions has made this process increasingly competitive. This trend has further underscored the need for further research in the fellowship selection process.

Specifically, concerning the arthroplasty literature, there is little information available on the criteria used to select candidates, character traits that may influence an individual to gravitate toward arthroplasty, and characteristics that make a successful arthroplasty surgeon. Such data would be useful to selection committees and candidates alike [4].

Therefore, the purpose of this study is to identify qualities among arthroplasty applicants to our institution that correlated with their final position on our institution’s rank list. For the 2017 application cycle, all 40 applicants who applied for the fellowship program were invited for the interview. We hypothesize that the interview portion of the assessment is the most important factor in ranking, with little impact from other variables, such as the United States Medical Licensing Examination (USMLE) Step Scores, research experience, and caliber of medical and undergraduate training.

## Materials and methods

This is a cohort study analyzing the criteria for fellowship applications that correlate with the best matching at the Brown University Comprehensive Adult Reconstruction Fellowship. At our institution, all 40 applicants who applied were interviewed and evaluated for a fellowship position in adult reconstruction, and their applicant data and rankings were analyzed retrospectively.

The criteria evaluated included the following characteristics commonly considered to be of value when assessing applicants for fellowship: (1) perceived quality of undergraduate education; (2) perceived quality of medical school education; (3) perceived caliber of residency training; (4) USMLE Step 1, 2, and 3 scores; (5) quality of recommendation letters; (6) quality of the personal statement; (7) research experience; (8) extracurricular activities; and (9) performance on the interview day. Five fellowship-trained arthroplasty surgeons were involved in application screening and interviews. This resulted in five independent scores for each characteristic. Scores on each subsection were tabulated and used to create an objective rank list. The list was then adjusted to create a final interview rank list based on input and subjective feedback from each interviewer immediately after the interview day.

The perceived quality of education and training was based on the academic caliber of the institution and the applicants’ reputation at the national level, as scored by the US News and World Report. Applicant research was scored from 0 to 3, with a score of 0 given to applicants with no research, 1 to those with some research that may or may not have resulted in 1-2 publications, 2 for those with 3-10 publications, and 3 for those with >10 publications. Journal articles and book chapters were counted as publications, while abstracts and presentations were excluded. The personal statement section was scored on a scale of 1-3, with 1 given to a statement that was poorly written and difficult to comprehend and follow, and 3 to a well-written and organized statement which included good support for matching into a position in joint arthroplasty and details of unique personal experiences and traits supporting the application. Letters of recommendation were the sum of three scores ranging from 0-3, with a score coming from each of the three letters that an applicant submitted; hence, the maximum score an applicant could get was 9. A score of 0 was a letter that was not in support of the candidate, 1 was in support with reservations, 2 was supportive but not specific as to why, and a score of 3 was given to letters that demonstrated elements of enthusiasm.

Extracurricular activities included specifics regarding hobbies and experiences documented in the application and taken into account during the interview. Additionally, the performance at the interview represented perceived social skills under high-stress conditions and was weighted the heaviest at 30% of the entire application.

All the data were tabulated and analyzed using simple regression as well as analysis of variance test, with a p-value of <0.05 considered significant.

## Results

All 40 applicants were present on the interview day. Independent scores for interviews, research, letters of recommendation, and personal statements were assigned by five fellowship-trained arthroplasty surgeons. Most applicants matched into adult reconstruction and joint arthroplasty fellowship; eight of the 40 individuals did not match. The eight individuals who did not match at our institution were in the bottom 25% of the applicant pool based on our ranking system. The bottom five applicants were eliminated from the final rank list even if the interview, research, letters of recommendation, and personal statement categories scored well (Table 1). In addition, the applicants at the bottom of our ranking system did not match to a fellowship program in general, not just at our institution.

**Table 1:** The characteristics of the bottom five applicants. The applicant characteristics for the bottom five ranked applicants based on multiple criteria including USMLE Step 1, Step 2, and Step 3 scores, as well as values assigned, as discussed in the Materials & Methods section for the strength of recommendation letters and research. USMLE: United States Medical Licensing Examination

Medical School	USMLE Step 1	USMLE Step 2	USMLE Step 3	Letters	Research
70 to 79	250–259	260–269	210–219	8	3
Unranked	240–249	250–259	220–229	7	1
Canadian	200–209	220–229	220–229	7	2
70 to 79	230–239	200–219	210–219	5	1
91 to 120	220–229	230–239	210–219	8	0

**Figure 1:**
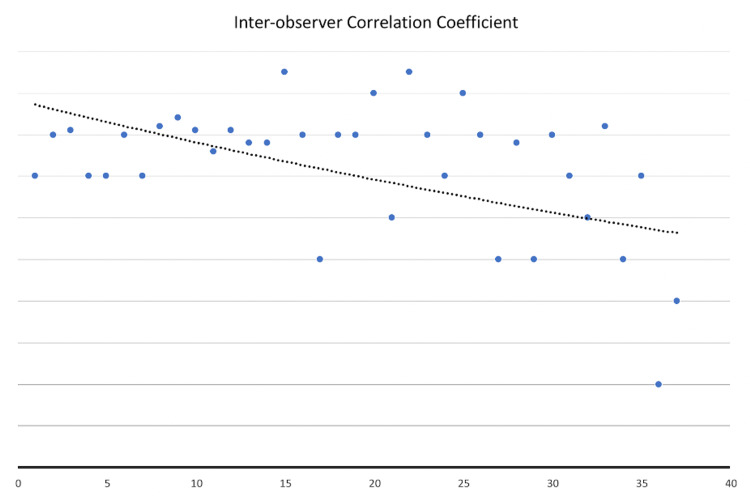
Intraclass correlation for interviewer scores for each applicant.

Despite the attempt at objective ranking, the position of 29% of applicants changed on the preliminary rank list after post-interview discussions. The majority of changes were related to applicants with the highest inter-observer variability. Although the interviewers provided their own unique rating after each interview, overall scores for each applicant were similar between the three interviewers (average intraclass correlation = 0.725). However, correlations were more consistent in the top 15 applicants and worse in the bottom group (Figure 1).

Three applicants who moved to the top 10 group had similar inter-observer scores. Applicant characteristics for the top five applicants are shown in Table 2.

**Table 2:** The characteristics of the top five applicants. Applicant characteristics for the top five ranked applicants based on multiple criteria including USMLE Step 1, Step 2, and Step 3 scores, as well as values assigned, as discussed in the Materials & Methods section for the strength of recommendation letters and research. USMLE: United States Medical Licensing Examination

Medical school	USMLE Step 1	USMLE Step 2	USMLE Step 3	Letters	Research
Unranked	240–249	250–259	230–239	8	3
30 to 39	240–249	220–229	220–229	6	1
50 to 59	230–239	230–239	210–219	8	2
10 to 19	260–269	260–269	240–249	6	2
Unranked	210–219	220–229	200–209	6	0

**Figure 2:**
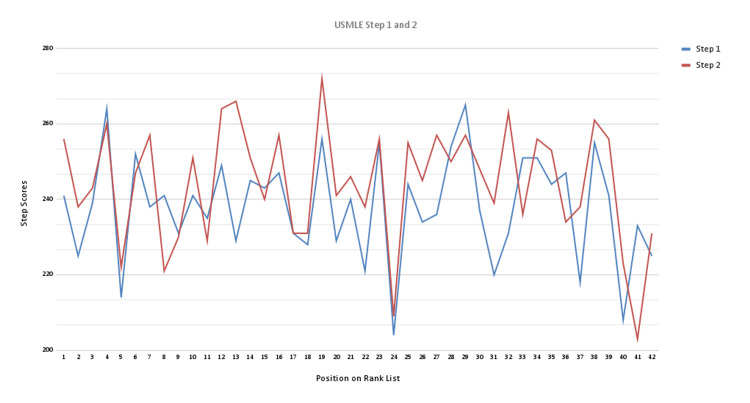
Correlation between USMLE Step 1 (blue) and Step 2 (red) scores and the final position on our institution’s rank list. USMLE: United States Medical Licensing Examination

Of the variables assessed, the only significant factor associated with a higher position on the final rank list was the combined interview score with a correlation coefficient of 0.92. Within the interview score, the most important components were subjective indications of (1) personality and (2) fit with the program. USMLE Step 1 and 2 scores had relatively lower correlation coefficients at 0.32 and 0.29, respectively (Figure 2).

Although letters of recommendation had a relatively low correlation coefficient at 0.2, they were helpful in eliminating concerning applicants. The personal statement and research components demonstrated the lowest correlations. Although the caliber of the medical school correlated with a higher rank list position than the caliber of the applicant’s residency or undergraduate institution, the correlations for these were generally very low.

## Discussion

There has been a recent trend towards increased subspecialization within orthopedics, with the majority of current trainees pursuing fellowship training after residency [5-10]. Concurrently, there has been an increasing demand for arthroplasty procedures, as well as a need for fellowship-trained arthroplasty surgeons to supply this demand [11-13]. Unfortunately, there is a paucity of literature guiding arthroplasty fellowship applicants and fellowship directors in the fellowship match process. The purpose of this study was to identify qualities in arthroplasty fellowship applicants at our institution which correlated with their final position on the rank list. Our findings demonstrate that at our institution personality and individual fit were the most important determinants of successful ranking in the fellowship match.

During the 2017 application cycle, all 40 residents who applied for the arthroplasty fellowship at our institution were selected for an interview. This factor makes our study unique because there was no pre-interview selection based upon other commonly assessed variables such as the USMLE step scores, research, or the caliber of the residency program. Evaluation and ranking of applicants were not completed until after the interview at our institution.

The combined interview score was the only statistically significant variable that correlated with higher ranking, with the most important subcomponents being subjective indications of applicant personality and fit within the program. This is consistent with previous survey-based studies of orthopedic fellowship program directors, which found that the interview was the most important factor in determining an applicant’s rank [3,14,15].

Similarly, the relative importance of the interview has also been demonstrated in the literature regarding non-orthopedic specialty fellowships [16-22]. Although personality and program fit are important factors for residency, this component can be even more important in fellowship given the close daily interaction of fellows with mentors throughout the fellowship year. Previous studies have demonstrated that the fellowship interview process is a significant burden on both fellowship applicants and residency programs [23,24]. Given the relative importance of the interview in the fellowship match process, there may be a need to explore alternative methods of assessing qualities such as personality and individual fit [25].

Interestingly, recommendation letters, personal statements, USMLE scores, research components, and caliber of undergraduate school, medical school, and residency program had relatively low correlations with ranking. This is in contrast with previous studies that have shown some of these factors, in particular recommendation letters, to be relatively important in the fellowship match process [17,18,26]. Given that the majority of recommendation letters are positive, this component likely has poor discriminatory ability in differentiating candidates once they are selected for the interview. Because all applicants at our institution were selected for interviews, the low correlation of recommendation letters in the ranking of applicants at our institution follows this theory. Regarding USMLE scores, research productivity, and caliber of institution, these objective measures of performance are likely less useful once applicants have reached this later stage in their medical career where subjective qualities such as personality and interpersonal skills become increasingly valued.

Although the interview scores were subjective and there were no set thresholds or score minimums used to eliminate applicants, we found that this highlights an important finding of this study. Because all applicants were interviewed, no one was eliminated based on standardized testing scores, research experience, or other factors, allowing us to find the best candidates who fit our program and ensuring that good candidates were not disqualified based on a low score in a singular category. This allowed us to holistically analyze each applicant, compare notes with other members of the interview committee, and create a final rank list that took into account all the above-mentioned factors. Additionally, this allowed us to see that research experience, USMLE Step scores, and other traditional variables that are assessed for fellowship matching did not have much impact on the final applicant rank (Figure 2). Because no applicants were eliminated from the interview process, we were able to analyze every factor without worrying about having an incomplete dataset.

There are limitations to this study. This is a single institution and fellowship program perspective, which is likely to differ from other programs in the country. There is a wide range of program characteristics such as geographical location, case volume and variety, differences in surgical exposures and techniques, and variations in academic productivity, which may influence both the types of applicants who apply to and are selected by the program. Furthermore, the list of evaluated application characteristics was not exhaustive, and other subjective factors such as personal connections between applicants and programs were not evaluated. Our methods for quantifying arguably subjective aspects of an application such as research, personal statements, letters, extracurricular activities, and quality of education have not been validated. Perhaps most importantly, the predictive validity of each of these metrics in assessing the future success of individual applicants in fellowship and beyond remains unclear.

## Conclusions

Overall, this study aims to fill a gap in the literature by providing comprehensive empirical data from an entire cohort of fellowship applicants to an adult reconstruction fellowship program. This information may help guide future fellowship applicants and provide a better understanding of the ranking process. Furthermore, the fellowship match is not a perfect process, and this data may help provide some insights into ways of improving the fellowship match process in the future. As expected, personality and program fit were the most important determinants of successful ranking at our institution. Ultimately, the ranking does not correlate with the applicant’s success in fellowship, and more work is needed to determine the qualifications of a good surgeon.
